# Telomere length is associated with oppositional defiant behavior and maternal clinical depression in Latino preschool children

**DOI:** 10.1038/tp.2015.71

**Published:** 2015-06-16

**Authors:** J M Wojcicki, M B Heyman, D Elwan, S Shiboski, J Lin, E Blackburn, E Epel

**Affiliations:** 1 Department of Pediatrics, University of California, San Francisco, San Francisco, CA, USA; 2 Department of Epidemiology and Biostatistics, University of California, San Francisco, San Francisco, CA, USA; 3 Department of Biochemistry and Biophysics, University of California, San Francisco, San Francisco, CA, USA; 4 Department of Psychiatry, University of California, San Francisco, San Francisco, CA, USA

## Abstract

Exposure to psychological stress and depression are associated with shorter white blood cell telomere length (TL) in adults, possibly via associated lifelong oxidative stressors. Exposure to maternal depression increases risk for future depression and behavior problems in children, and Latino youth are at high risk. Few studies have evaluated the role of exposure to maternal depression or child behavior in relation to TL in children. We assessed early-childhood exposures to maternal depression from birth to the age of 5 years and child behavior from ages 3–5 years in a cohort of Latino children in relation to child leukocyte TL at ages 4 and 5 years. Children who had oppositional defiant behavior at 3, 4 or 5 years had shorter TL than those without by ~450 base pairs (*P*<0.01). In multivariate analyses, independent predictors for shorter TL at 4 and 5 years of age included oppositional defiant disorder at 3, 4 or 5 years (*β*=−359.25, 95% CI −633.84 to 84.66; *P*=0.01), exposure to maternal clinical depression at 3 years of age (*β*=−363.99, 95% CI −651.24 to 764.74; *P*=0.01), shorter maternal TL (*β*=502.92, 95% CI 189.21–816.63) and younger paternal age at the child's birth (*β*=24.63, 95% CI 1.14–48.12). Thus, exposure to maternal clinical depression (versus depressive symptoms) in early childhood was associated with deleterious consequences on child cellular health as indicated by shorter TL at 4 and 5 years of age. Similarly, children with oppositional defiant behavior also had shorter TL, possibly related to early exposures to maternal clinical depression. Our study is the first to link maternal clinical depression and oppositional defiant behavior with shorter TL in the preschool years in a relatively homogenous population of low-income Latino children.

## Introduction

Telomeres are the protective DNA and protein complexes at the end of chromosomes and ensure chromosomal stability. Shorter telomere length (TL) is associated with aging and aging-related diseases and conditions including obesity, diabetes and cancer.^1, 2, 3, 4^

### Telomeres and exposure to psychological stress and depression

Exposure to psychological distress including clinical depression is associated with shorter TL in adults.^5, 6^ Data from studies of adult caregivers exposed to chronic stress and those with chronic depression, including remitted major depressive disorders, have shorter TL in comparison with controls.^7, 8^ Telomere shortening in chronically depressed adult individuals corresponds to ~7 years of accelerated cell aging.^9^ Leukocyte telomere shortening through exposure to chronic stress can result in immune senescence and impaired leukocyte function, which is thought to create a vicious cycle of inflammatory cytokine output and oxidative stress.^9^ Chronic stress could also lead to a greater expression of latent viruses such as Epstein–Barr virus^10^ or cytomegalovirus, which have been implicated in telomere shortening.^11^ Post-traumatic stress disorder in adults has also been associated with apparent acceleration of aging, with those individuals with post-traumatic stress disorder having significantly shorter leukocyte TL.^12^

Early childhood, including the prenatal period is characterized by increased epigenetic modeling.^13^ Epigenetic processes such as methylation have been reported to be altered by exposure to early adversity including child abuse and prenatal depression.^14, 15^ Several studies have linked shorter adult leukocyte TL to early-childhood trauma or adversity including maltreatment, exposure to low income, low maternal education and unstable family structure.^16, 17, 18, 19^ A recent study reported that shorter TL at 5–10 years of age is associated with exposure to violence in childhood.^20^ A study of TL of Romanian children in middle childhood found that children with the greatest exposure to institutional care during early childhood had the shortest telomeres,^21^ and a recent study also found shorter TL associated with psychological stress exposure, as indicated by adverse neighborhood environment.^22^

Although there are many studies of depression and stress in adulthood and environmental exposures in childhood, few studies have specifically investigated childhood behavior and TL, or the combined effects of exposure to maternal depression and child behavior problems on TL in high-risk children.^23, 24^ Early childhood is a crucial time period to better understand TL and future risk for chronic disease, as the rate of attrition is steepest in the first 5 years of life and likely sets the course for future processes of telomere attrition.^25^ In addition, although it is well known that early exposures to adversity and maternal depression increase risk for future psychiatric disorders and chronic disease risk, the mechanistic pathways for these relationships are not well understood.^26, 27, 28, 29^ Latino children are at higher risk for a number of chronic health conditions including behavior issues in childhood^30^ and depression,^31^ so having a better understanding of relationship between maternal and child factors resulting in shorter TL in the preschool years could shed light on the role of early-life risk factors for future health trajectories.

We evaluated the effect of exposure to maternal depression (symptoms and clinical depression) and child behavior problems on TL in early childhood in a group of high-risk, low-income Latino children followed from birth in San Francisco, California.

## Materials and methods

### Telomere length

We examined TL by quantitative PCR using genomic DNA from dried blood spots in a sample of 108 4-year-old, low-income Latino children and their mothers and 92 5-year-old children, with 3 children having 3 different samples collected over the 2-year period for a total of 203 samples collected. Each mother had only one child with telomeres analyzed. TL is expressed as *T*/*S* (the ratio of telomeric product versus single-copy gene product). DNA was extracted using the QIAamp DNA Investigator Kit (Qiagen, Hilden, Germany, Cat # 56504). The TL measurement assay is adapted from the published original method by Cawthon.^32, 33^

To control for inter-assay variability, 8 control DNA samples are included in each run. In each batch, the *T*/*S* ratio of each control DNA is divided by the average *T*/*S* for the same DNA from 10 runs to get a normalizing factor. This is done for all eight samples and the average normalizing factor for all 8 samples is used to correct the participant DNA samples to get the final *T*/*S* ratio. The *T*/*S* ratio for each sample is measured twice. When the duplicate *T*/*S* value and the initial value vary by more than 7%, the sample is run the third time and the two closest values are reported. The average closest value for this study is 4.8%.

To convert *T*/*S* ratios to base pairs, the above method is used to determine the *T*/*S* ratios of a set of genomic DNA samples from the human fibroblast primary cell line IMR90 at different population doubling, as well as with the telomerase protein subunit gene hTERT infected on a lentiviral construct. This set of DNA samples represents different *T*/*S* ratios from the same parental cell line. The mean telomeric restriction fragment length from these DNA samples is determined using Southern blot analysis and compared with the *T*/*S* ratios for these samples to convert *T*/*S* ratios to base pairs. This was expressed as the following formula: base pairs=3274+2413 × (*T*/*S*).

### Cohort and procedures

This group of children and their mothers were recruited prenatally at two hospitals in San Francisco in 2006–2007 at which time sociodemographic and health history was assessed including maternal depression. The cohort has been described in previous publications including recruitment, inclusion and exclusion criteria and data collection.^34, 35, 36^ Sample size calculations were initially done for the relationship between maternal depressive symptoms and child weight gain. Briefly, child weight and length and maternal body mass index have been assessed annually from birth with the child's weight and length measured also at birth and 6 months of age. Maternal depressive symptoms were assessed prenatally, at 4–6 weeks postpartum and annually throughout the follow-up period until age 5 years using the Edinburgh Postpartum Depression Scale^37^ and the Center for Epidemiologic Studies Depression Scale^38^ to assess for depressive symptoms, and the Mini International Neuropsychiatric Interview (MINI, version 5.0)^39^ was used to evaluate for current major depressive episodes. All interviews were conducted either in either English or Spanish and all measures to assess mental health used had previously been validated in Spanish speaking populations as previously described.^34, 35, 36^ The study was approved by the Institutional Review Board (the Committee on Human Research) at the University of California, San Francisco. All mothers provided written consent for their participation and their children's participation.

Child psychiatric and behavior problems, specifically internalizing and externalizing behaviors, were assessed at 3, 4 and 5 years of age using the Child Behavior Checklist (CBCL/1½-5), which is comprised of validated preschool items that experienced child psychiatrists and psychologists from 16 different cultures rated as being most consistent with clinical Diagnostic and Statistical Manual of Mental Disorders (DSM) diagnostic categories.^40^

### Predictor and outcome variables

#### Depression

Exposure to pre- or postnatal depressive symptoms were defined by: (1) Center for Epidemiologic Studies Depression Scale ⩾16; (2) Edinburgh Postpartum Depression Scale ⩾13; or (3) having a major depressive episode or dysthymia as per the MINI. A high depression symptom score was defined as a high score on one of the above measures prenatally or at the 4–6-week, 6-month, 1–5-year time points. Chronic depression was defined as having a high score at multiple time points or >2 time points using the following categorizations (1) prenatal to 4–6 weeks postpartum, (2) prenatal to 5 years postpartum or (3) 3–5 years postpartum).

#### Child behavior

Independent research studies have validated CBCL syndrome scales in over 30 societies^40, 41, 42^ and include affective, anxiety, pervasive developmental, attention deficit/hyperactivity and oppositional defiant problems. For each participant, *T*-scores and percentiles were calculated using CBCL/1½-5 scoring guidelines. *T*-scores that were >65 for any given DSM-oriented scale were identified as in the 'clinical range' per scoring guidelines.^40^ We also created new variables at each time point to assess the possible relationship between any psychopathology (having one or more of affective, anxiety, developmental or oppositional defiant problems at 3, 4 or 5 years of age). Finally, because of the statistical associations found between oppositional defiant behavior and TL at 3 and 4 years of age and approaching statistical significance at 5 years, but the low number of children with these conditions, we created a new variable assessing any oppositional defiant behavior at 3, 4 or 5 years of age.

### Statistical analysis

We used linear regression models to investigate the relationship between TL and categorical predictors of maternal depressive symptoms, maternal clinical depression and child clinical behavior outcomes (attention, anxiety, affective, developmental and oppositional). Fitted models adjusted for age of telomere collections, and associated inferences accounted for repeat telomere measurements for some children using robust s.e. To account for the possible impacts of departures from normality on our inferences, alternate regression models based on the gamma distribution were fitted. All analyses were conducted using Stata 13.0 (StataCorp, College Station, TX, USA).

Initial analyses focused on marginal associations between predictor variables and TL, with results summarized as group-specific mean±s.e. Variables displaying significant associations with TL (*P*<0.05) were considered in regression models, including multiple predictors adjusting for known predictors of child TL including maternal TL and father's age at birth.

As child anxiety at 5 years of age and oppositional defiant behavior were highly correlated with one another, these two variables were not analyzed in the same multivariate model. Rather, we ran two separate multivariate models that assessed the associations for oppositional defiant behavior and child anxiety separately, adjusting for child's age at collection of telomeres, maternal TL and paternal age at birth. Because of the high number of missing data points from paternal age (~18%), we ran two multivariate models comparing results with and without paternal age. We performed additional analyses to assess the possible role of child oppositional defiant behavior as a mediator of the relationship between exposure to clinical maternal depression at age 3 years and shorter TL at ages 4 and 5 years. These were based on methods for causal mediation analysis,^43^ and are summarized as the estimated percentage of the effect of maternal depression on TL that may be explained by mediation. Finally, we assessed the associations between clinical depression at 3 years of age and child anxiety and oppositional defiant behavior using logistic regression models to better understand possible relationships between maternal and child mental health in relation to child telomere outcomes at ages 4 and 5 years.

## Results

### Maternal depression

For the different time points, the percentage of infants with mothers who had depressive symptoms ranged from 32.1% (65/203) in the prenatal period to 12.0% (23/191) at 12 months of age. We did not find significant associations between child TL and exposure to maternal depressive symptoms or maternal clinical depression examining this relationship in the prenatal, postnatal (4–6 weeks, 6 months and 12 months) and early-childhood period (3, 4 and 5 years), with the exception of a statistically significant relationship between exposure to maternal clinical depression at 3 years of age and shorter TL of ~450 base pairs (7350.67±155.67 versus 7795.20±64.00, *P*<0.01) (Table 1). We did not find any relationship between chronic maternal depressive symptoms and shorter TL comparing children who had been exposed to chronic depression from birth through 5 years of age (*P*=0.88) and those who had been exposed from 3–5 years of age (*P*=0.47). The number of mothers who had chronic, clinical depression from 4–5 years postpartum was very small (*n*=3), although the mean TL in those children was much shorter than in unexposed children (7192.97±101.64 versus 7821.54±67.84 for children of mothers with clinical depression at 4–5 years of age; *P*<0.01). However, because the numbers were so small, we were unable to conduct multivariate analyses, adjusting for possible cofounders, in this subsample. We also do not present these data in tabular format because of the small sample size and inability to make any conclusions or conduct analyses.

### Child behavior

**Figure 1:**
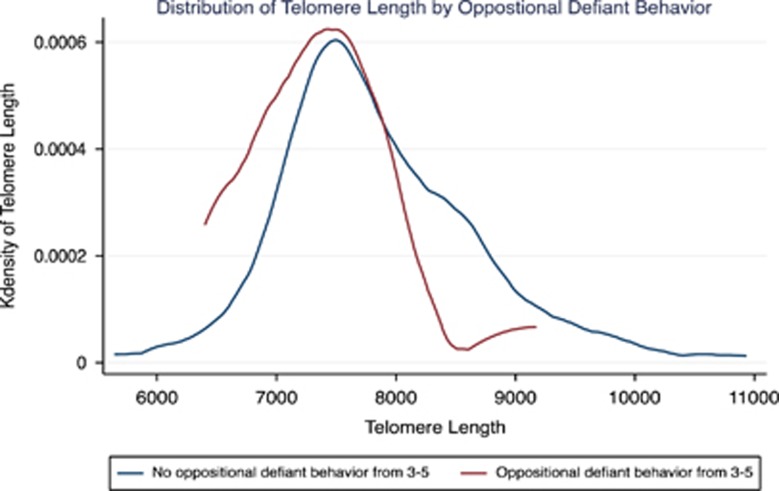
The frequency of telomere length for those with any oppositional defiant behavior versus none. The x axis is telomere length in base pairs and y axis is probability with the area under the curve representing 1.

We found statistically significant associations between child behavior at 3, 4 and 5 years of age and TL. Child anxiety at 5 years of age was associated with reduced TL by ~350 base pairs (7479.73±97.07 versus 7818.05±69.32; *P*<0.01). At 4 years of age, TL was also slightly but not statistically significantly lower in those children with anxiety (7552.01±164.40 versus 7823.48±69.62; *P*=0.13). Children with oppositional defiant behavior at 3 and 4 years had shorter TL compared with those without oppositional defiant behavior (6981.04±336.02 versus 7816.29±66.83; *P*=0.02 for 3 years and 7401.84±155.20 versus 7822.30±68.69; *P*=0.01 for 4 years; Table 2). Children who had oppositional defiant behavior at 3, 4 or 5 years (11/117 or 9.4%) also had shorter TL than those without (7366.03±176.64 versus 7847.69±71.27, or approximately a 500-base-pair difference (*P*=0.01) (Figure 1). We were unable to examine the relationship between chronic child behavior issues and TL because of the small sample size in the groups with chronic behavior issues beyond one time point.

#### Multivariate analysis

We conducted a multivariate analysis for independent predictors of shorter TL at 4 and 5 years of age controlling for possible confounders including paternal age at the child's birth, child sex and adult TL and exposure to maternal clinical depression at 3 years of age and oppositional defiant behavior at 3, 4 or 5 years of age. Independent predictors for shorter TL included maternal clinical depression at 3 years of age (*β*=−363.99, 95% CI −651.24 to 764.74; *P*=0.01), younger paternal age (*β*=24.63, 95% CI 1.14–48.12; *P*=0.04) and shorter maternal TL (*β*=502.92, 95% CI 189.21–816.63; *P*<0.01) (Table 3). In a model without paternal age but a greater number of observations (*n*=180), predictors of shorter TL were comparable. After adjusting for other predictors, child anxiety at 5 years of age was no longer associated with shorter TL (*β*=−41.27, 95% CI −369.32 to 286.79; *P*=0.65). In an additional analysis, maternal clinical depression at 3 years of age was associated with child clinical anxiety problems at 5 years of age (odds ratio 9.0, 95% CI 1.89–42.96) and oppositional defiant behavior at 3 years of age (odds ratio 15.44, 95% CI 1.20–198.52), although maternal clinical depression was not associated with oppositional defiant problems at other time points. We did not find evidence of significant mediation by child oppositional defiant behavior in explaining the relationship between shorter TL and maternal clinical depression at age 3 years.

## Discussion

We found that that maternal clinical depression at 3 years of age was associated with shorter TL at 4 and 5 years of age by ~450 base pairs. This relationship was still statistically significant after controlling for maternal TL, paternal age and child anxiety at 5 years and oppositional defiant behavior at 3, 4 or 5 years. We also found that chronic maternal clinical depression from 4 to 5 years of age was associated with significantly shorter TL, but the number of children whose mothers were chronically depressed was too small to make any conclusive statements. Further studies with larger cohorts are needed to verify our findings.

We believe ours is the first longitudinal study to evaluate the role of exposure to maternal depressive symptoms at different time points in early childhood and telomere shortening, including prenatal and early-childhood exposures. Similar to other cross-sectional studies that have found an association between exposure to adversity and shorter TL,^20, 21, 22^ we found that in our relatively homogenous, low-income Latino cohort, shorter TL was associated with more recent exposures to more severe maternal clinical depression. We did not find any evidence that maternal depressive symptoms, in contrast to clinical depression, were associated with shorter child TL. Similarly, a recent study by Gotlib *et al.*^44^ found that older girls (approximately 11–12 years) of mothers with a history of or current clinical depression (but not depressive symptoms) had shorter telomeres than girls of mothers without depression.

A recent study found that the telomeres are shorter in white matter oligodendrocytes of the prefrontal cortex in patients with major depressive disorder.^45^ Although many studies have focused on telomere shortening in leukocytes, this was the first study to show that telomere shortening occurs in brain tissues in major depressive disorder. Similar to leukocytes, oliogodendrocytes are sensitive to reactive oxygen species damage, and suggests white matter pathologies may be instrumental for clinical depression genesis or sustainment. We believe our study is the first study finding that maternal clinical depression is associated with young children's shorter TLs, although the mechanisms of these processes are not known. It is notable that the early-prenatal maternal clinical depression was not associated with shorter TL at age 4 and 5 years, but maternal clinical depression from 4 to 5 years of age was suggesting possibly environmental mechanisms.

Also unique to our study, although our sample size was limited we found evidence to support an association between child behavior problems, in particular, oppositional defiant behavior or anxiety and shorter TL at 4 and 5 years of age, similar to the study by Kroenke *et al.*,^24^ which found that highly 'stress-reactive' children with more internalizing behaviors had shorter TL. TL was also associated with high sympathetic activation, parasympathetic withdrawal and/or high cortisol response. Lower parasympathetic tone and withdrawal to a stressor has been shown to be associated with low levels of telomerase, the enzyme which helps maintain TL.^5^ However, in contrast with Kroenke *et al.*,^24^ we found an association between oppositional or externalizing behaviors, and shorter TL at 4 and 5 years of age. In logistic models, maternal clinical depression at 3 years of age was associated with oppositional defiant behavior at age 3 years and child anxiety at age 5 years, possibly suggesting a pathway between exposure to maternal clinical depression symptoms and shortening of child TL through development of behavioral problems. However, in mediation analysis we did not find any role for oppositional defiant behavior explaining the relationship between maternal clinical depression at 3 years and shorter TL in children. In addition, we do not have TL at birth and so we have no way of assessing whether children with shorter TL related to oppositional defiant behavior or child anxiety actually had shorter TL prior to the development of behavior problems.

A unique aspect of our cohort is that the sample is relatively homogenous in terms of race and ethnic background, levels of acculturation and socioeconomic status to the United States as previously described.^34, 35, 36^ For these reasons, we can be relatively confident that differences observed based on TL in relation to maternal clinical depression or child oppositional defiant behavior are not explained by differences in socioeconomic status, cultural background or other unmeasured confounders. Given the high rates of depression, anxiety and behavior problems reported in Latino youth and adults,^30, 31^ this is an important population in which to better understand the relationship between inter-generational transmission of psychiatric and behavior problems.

A limitation of our study, in addition to the small sample size and limited number of outcomes for some of the predictors, is that we only measured TL twice over a short time period (1 year). Future studies should begin telomere collection at birth and evaluate predictors sequentially in relation to changes in telomere over time.

## Figures and Tables

**Table 1:** Maternal depressive symptoms and clinical depression in relation to child telomere length at 4 and 5 years of age

*Time point in* *pregnancy/childhood*	*Telomere length (base pairs)±s.e.*					
	*Maternal depressive* *Sx* N/T^a^ *(%)*	P-*value*	*Maternal clinical* *depression* N/T^a^ *(%)*	P*-value*	*Maternal chronic* *depressive Sx* N/T^a^ *(%)*	P*-value*
*Prenatal*						
Yes	7879.79±129.32	0.44	7629.47±145.00	0.25		
	65/203 (32.1)		21/203 (10.3)			
No	7762.35±76.38		7819.04±72.14			
	138/203 (68.0)		182/203 (89.7)			
						
*4*–*6 Weeks*						
Yes	7828.67±137.92	0.82	7974.04±218.69	0.42		
	42/203 (20.7)		12/203 (5.9)			
No	7792.08±76.00		7788.65±69.26			
	161/203 (79.3)		191/203 (94.1)			
						
*6 Months*						
Yes	8082.31±162.93	0.09	7792.60±149.27	0.82		
	34/182 (18.7)		17/182 (9.3)			
No	7770.15±79.62		7830.28±78.35			
	148/182 (81.3)		165/182 (90.7)			
						
*12 Months*						
Yes	7828.05±165.23	0.94	7743.67±203.39	0.71		
	23/191 (12.0)		17/191 (8.9)			
No	7814.96±75.77		7823.68±73.86			
	168/191 (88.0)		174/191 (91.1)			
						
*3 Years*						
Yes	7571.43±137.08	0.15	7350.67±155.67	**<0.01**		
	29/192 (15.1)		14/194 (7.2)			
No	7794.28±68.15		7795.20±64.00			
	163/192 (84.9)		180/194 (92.8)			
						
*4 Years*						
Yes	7926.05±192.77	0.50	7550.49±370.63	0.47		
	31/193 (16.1)		13/196 (6.6)			
No	7785.83±73.89		7827.03±68.14			
	162/193 (83.9)		183/195 (93.4)			
						
*5 Years*						
Yes	7890.17±181.25	0.56	7707.41±151.61	0.54		
	38/195 (19.5)		19/195 (9.7)			
No	777.86±66.35		7810.11±69.68			
	157/195 (80.5)		176/195 (90.3)			
						
*Prenatal* *4–6 weeks*						
Yes					7817.26±181.68	0.92
					10/203 (4.9)	
No					7798.66±69.04	
					193/203 (95.1)	
						
*Prenatal*—*5 years*						
Yes					7754.4±299.63	0.88
					4/203 (2.0)	
No					7800.39±67.48	
					199/203 (98.0)	
						
*3*–*5 Y*ears						
Yes					7597.34±301.35	0.47
					12/200 (6.0)	
No					7822.32±68.56	
					188/200 (94.0)	

Abbreviation: Sx, symptoms.

a *N*/*T* is the number of observations over the total assessed for that variable. All analyses presented in the table are unadjusted.

*P*-values <0.01 are highlighted in bold.

**Table 2:** Child behavior and child telomere length at 4 and 5 years of age

*Child behavior issue*	*Telomere length (base pairs)±s.e.*					
	*3 Years of age* N/T*a (%)*	P-*value*	*4 Years of age* N/T*a (%)*	P*-value*	*5 Years of age* N/T*a (%)*	P-*value*
*Attention*						
Yes	7895.25±382.10	0.80	8080.13±184.08	0.17	7699.68±283.86	0.71
	8/200 (4.0)		7/198 (3.5)		6/198 (3.1)	
No	7795.62±67.80		7808.19±68.85		7809.27±68.80	
	192/200 (96.0)		191/198 (96.5)		192/198 (97.0)	
						
*Anxiety*						
Yes	7803.21±147.18	0.98	7552.01±164.40	0.13	7479.73±97.07	**<0.01**
	24/200 (12.0)		7/198 (3.5)		7/198 (3.5)	
No	7799.14±73.44		7823.48±69.62		7818.05±69.32	
	176/200 (88.0)		191/198 (96.5)		191/198 (96.5)	
						
*Affective*						
Yes	8078.77±213.19	0.18	7612.55±177.97	0.27	7762.69±211.99	0.84
	13/200 (6.5)		7/198 (3.5)		9/198 (4.6)	
No	7780.14±69.14		7821.24±69.54		7808.15±69.58	
	187/198 (93.5)		191/198 (96.5)		189/198 (95.5)	
						
*Developmental*						
Yes	7726.13±131.30	0.56	7860.86±207.22	0.82	7564.53±225.30	0.28
	36/200 (18.0)		12/198 (6.1)		5/198 (5.1)	
No	7816.06±76.23		7810.59±70.64		7819.44±69.30	
	164/200 (82.0)		186/198 (93.9)		188/198 (95.0)	
						
*Oppositional defiant*						
Yes	6981.04±336.02	0.02	7401.84±155.20	0.01	7519.63±198.91	0.15
	4/200 (2.0)		4/198 (2.0)		10/202 (5.0)	
No	7816.29±66.83		7822.30±68.69		7820.43±68.76	
	196/200 (98.0)		194/198 (98.0)		192/202 (95.0)	

a *N*/*T* is the number of observations over the total assessed for that variable. All analyses presented in the table are unadjusted.

*P*-values <0.01 are highlighted in bold.

**Table 3:** Multivariate analysis of predictors for shorter telomere length at 4 and 5 years (*n*=151)^a^

*Predictor variables*	β *(95% CI)*	P*-value*
Maternal telomere length	502.92 (189.21 to 816.63)	<0.01
Paternal age at child's birth	24.63 (1.14 to 48.12)	0.04
Maternal clinical depression at 3 years of age	−363.99 (−651.24 to 764.74)	0.01
Child oppositional defiant behavior at 3, 4 or 5 years of age	−359.25 (−633.84 to 84.66)	0.01
Child age at telomere collection	6.80 (−5.67 to 19.25)	0.28
Child sex (male)	−72.76 (−347.74 to 202.22)	0.60

a All variables in the table have been adjusted for in multivariate analyses.

## Notes

JL is a consultant to Telomere Diagnostics Inc., formerly Telomere Health, and owns stock in the company, and the company did not play any role in this research. The remaining authors declare no conflict of interest.
